# Construction of SGA prediction model based on multi-dimensional indicators in the second trimester of pregnancy: integrating parturient characteristics, serum markers and ultrasound parameters

**DOI:** 10.3389/fped.2025.1655615

**Published:** 2025-09-30

**Authors:** Shuying You, Shaohui Chen, Jing Gu, Shaolin Peng, Wenji Liu

**Affiliations:** ^1^ Department of Obstetrics, The Sixth Affiliated Hospital of Jinan University, Dongguan, Guangdong, China; ^2^ Department of Ultrasound, The Sixth Affiliated Hospital of Jinan University, Dongguan, Guangdong, China

**Keywords:** small for gestational age, prediction model, maternal baseline characteristics, first-trimester indicators, second-trimester fetal ultrasound indicators

## Abstract

**Objective:**

To develop and validate a prediction model integrating first-trimester maternal characteristics, serum markers, and second-trimester fetal ultrasound parameters for small-for-gestational-age (SGA) infants.

**Methods:**

This retrospective study analyzed 546 pregnant women (training set: *n* = 382; validation set: *n* = 164) from February 2022 to December 2024. Maternal baseline data, first-trimester pregnancy-associated plasma protein-A (PAPP-A) and *β*-human chorionic gonadotropin (*β*-hCG) levels, and second-trimester ultrasound indicators were collected. Multivariate logistic regression identified independent predictors, and a nomogram was constructed. Model performance was assessed using receiver operating characteristic (ROC) curves, calibration plots, and decision curve analysis (DCA).

**Results:**

The incidence of SGA was 18.85% (72/382) in the training set and 19.51% (32/164) in the validation set. Multivariate logistic regression showed that maternal age, levels of PAPP-A and *β*-hCG in the first trimester, fetal abdominal circumference, femur length, and umbilical artery PI in the second trimester were independent risk factors for SGA (all *P* < 0.05). The nomogram model showed good calibration and predictive efficacy in both the training set and the validation set. The C-index reached 0.783 and 0.754 respectively. The areas under the ROC curve (AUC) 0.783 [95% confidence interval (CI): 0.716–0.850] and 0.754 (95% CI: 0.641–0.867) respectively. The optimal thresholds determined based on Youden's index were 0.206 (sensitivity = 0.726, specificity = 0.745) for the training set and 0.227 (sensitivity = 0.747, specificity = 0.714) for the validation set.

**Conclusion:**

The nomogram prediction model constructed with these combined indicators is helpful for evaluating the risk of SGA. However, further verification through large-sample and multi-center studies is still needed to provide a reference for early clinical intervention.

## Introduction

Small for gestational age (SGA) refers to newborns whose birth weight is below the 10th percentile of the weight of fetuses of the same gestational age and gender (1). SGA is not only closely associated with perinatal adverse outcomes, such as neonatal asphyxia, hypoglycemia, polycythemia, etc., but may also increase the risk of developing chronic diseases such as cardiovascular diseases and metabolic syndrome in adulthood (2). Globally, the incidence of SGA ranges from 5% to 15%, and in China, epidemiological data show it is approximately 8%–12%, accounting for a significant proportion of perinatal adverse events. Clinically, SGA infants have a 3–5-fold higher risk of neonatal asphyxia, hypoglycemia, and polycythemia compared to appropriate-for-gestational-age infants; in the long term, they face a 1.5–2-fold increased risk of cardiovascular diseases (e.g., hypertension, coronary heart disease) and metabolic syndrome (e.g., type 2 diabetes) in adulthood. This substantial disease burden imposes heavy pressure on maternal and child health services and family care, underscoring the urgency of developing accurate SGA prediction tools (3). Accurately predicting SGA is of great significance for early intervention and improving the perinatal prognosis of mothers and infants. The maternal baseline characteristics of pregnant women, such as age, body mass index (BMI), underlying diseases, etc., have a significant impact on fetal growth and development (4). Pregnancy-associated plasma protein-A (PAPP-A) and β-human chorionic gonadotropin (β-hCG) in the first trimester of pregnancy are key markers secreted by the placenta, and changes in their levels can reflect placental function and the fetal growth environment (5). Fetal ultrasound indicators in the second trimester of pregnancy can intuitively and dynamically evaluate the status of fetal growth and development (6). In clinical management, current SGA prediction often relies on single indicators (e.g., isolated second-trimester ultrasound or first-trimester serum markers), leading to low prediction accuracy. This delays timely interventions (e.g., nutritional guidance, close fetal monitoring) for high-risk pregnant women, increasing the risk of adversed perinatal outcomes. From a public health perspective, unrecognized SGA cases contribute to increased neonatal hospitalization rates and long-term chronic disease prevalence, elevating healthcare costs (7). A study in Arch Gynecol Obstet noted that regions with insufficient SGA screening tools had a 20% higher neonatal intensive care unit admission rate for SGA infants. These clinical and public health challenges confirm the need for a multi-dimensional integrated prediction model (8). However, at present, studies on constructing prediction models to evaluate the risk of SGA by combining these multi-dimensional indicators are still insufficient. The development of this model and nomogram was driven by two key needs: Firstly, existing SGA prediction models rarely integrate maternal baseline characteristics, first-trimester serum markers, and second-trimester ultrasound parameters simultaneously, resulting in incomplete capture of SGA-related risk factors and limited prediction performance. Secondly, nomograms offer unique advantages in clinical practice—they transform complex regression equations into intuitive visual tools. Clinicians can quickly calculate an individual's SGA risk by summing scores of each indicator (e.g., maternal age, PAPP-A level) without relying on professional statistical software. This improves the accessibility and efficiency of risk assessment, especially in primary healthcare institutions with limited technical resources. In contrast, traditional regression models require complex calculations, making them difficult to apply in routine clinical work. Thus, developing a multi-dimensional nomogram is essential to bridge the gap between research and clinical practice. This study aims to deeply analyze the internal relationships between each indicator and SGA, construct and validate a precise prediction model, provide a more effective prediction tool for clinical practice, and improve the level of perinatal care.

## Materials and methods

### Study subjects

This retrospective study collected data from 546 pregnant women who received prenatal care and delivered at The Sixth Affiliated Hospital of Jinan University between February 2022 and December 2024. The participants were then randomly allocated to a training set (*n* = 382) and a validation set (*n* = 164) at a 7:3 ratio. Inclusion criteria were as follows: (1) Complete prenatal examination data during pregnancy, including detailed maternal baseline characteristics, first-trimester serological test results, and second-trimester fetal ultrasound examination reports. (2) Accurate gestational week records and singleton pregnancy. (3) The age of pregnant women ranged from 18 to 45 years old. Exclusion criteria were as follows: (1) Pregnant women complicated with severe dysfunction of important organs such as the heart, liver, and kidneys. (2) Pregnant women suffering from autoimmune diseases during pregnancy. (3) Fetuses with severe structural malformations or chromosomal abnormalities. (4) Use of drugs affecting fetal growth and development during pregnancy. This study was approved by the Ethics Committee of The Sixth Affiliated Hospital of Jinan University (Approval No. 2025-1-13). Written informed consent was obtained from all participants.

### Data collection

The maternal baseline characteristics of pregnant women were collected, including age, BMI, gestational weight gain, smoking history (defined as smoking ≥1 cigarette per day during pregnancy), drinking history (defined as drinking alcohol ≥1 time per week during pregnancy), history of hypertension, history of diabetes, history of pre-eclampsia, and family history of genetic diseases (defined as having a family member with a genetic disease).

Peripheral venous blood of pregnant women was collected in the first trimester (11–13 weeks), and the levels of PAPP-A and β-hCG were detected by chemiluminescence immunoassay.

In the second trimester (20–24 weeks), a comprehensive examination of the fetus was performed using a color Doppler ultrasound diagnostic instrument. Biparietal diameter (BPD), head circumference (HC), abdominal circumference (AC), femur length (FL), humerus length (HL), transverse cerebellar diameter (TCD), lateral ventricular width (LVW), and amniotic fluid index (AFI) were measured. Meanwhile, the ratio of peak systolic velocity to end-diastolic velocity (S/D) of the umbilical artery, the PI of the umbilical artery, and the fetal heart rate (FHR) were detected.

Two trained researchers independently reviewed and extracted data from electronic medical record system, laboratory information system, and picture archiving and communication system, and cross-validated the extracted information to ensure accuracy. Any discrepancies were resolved through discussion with a senior obstetrician.

### Model development and evaluation

In this study, a standard model development and validation process was adopted. Firstly, a nomogram prediction model was constructed based on the training set data. Corresponding scores were assigned to each independent risk factor in the model according to the results of multivariate logistic regression analysis. Then, the fitting effect of the model on the training set was evaluated by plotting the operating characteristic curve (ROC) curve, calibration curve, and decision curve analysis (DCA). These curves demonstrated the prediction ability and calibration performance of the model on the training data. Subsequently, the constructed model was applied to an independent validation set, and the generalization ability of the model was evaluated by the same evaluation methods (ROC curve, calibration curve, and DCA).

### Statistical analysis

Statistical analyses were performed using SPSS 26.0 and R 4.2.1. Count data were presented as the number of cases (percentage), and the *χ*^2^ test was used for comparisons between groups. Measurement data conforming to the normal distribution were presented as mean ± standard deviation, and the *t*-test was used for comparisons between groups. To ensure the reliability and generalization ability of the prediction model, we first divided the total sample (546 pregnant women) into a training set and a validation set using a computer-generated random sequence: the training set (*n* = 382, ∼70% of the total sample) was used for model construction, and the validation set (*n* = 164, ∼30% of the total sample) was used for model validation. Univariate analysis was used to screen possible influencing factors, and indicators with *P* < 0.05 were included in the multivariate logistic regression analysis to screen independent influencing factors [the results were presented as odds ratios (OR) with 95% confidence intervals (CI)], and variance inflation factors (VIF) were calculated to exclude multicollinearity (VIF threshold <10). A nomogram model was constructed based on the independent influencing factors. The model was internally validated using the Bootstrap method. The receiver ROC was used to evaluate the predictive efficacy of the model, and the area under the curve (AUC) and 95% CI were calculated. The calibration curve and Hosmer—Lemeshow test (*P* > 0.05 indicating good fit) were used to evaluate the consistency between the predicted values and the actual values. The DCA was used to evaluate its clinical application value. A *P* value < 0.05 was considered statistically significant. To clarify the validation process, a flow chart of the nomogram validation procedure is provided in Supplementary Figure S1, which details the steps from data collection to model evaluation.

## Results

### Comparison of general clinical characteristics of pregnant women in the training set and the validation set

A total of 546 pregnant women (training set: *n* = 382; validation set: *n* = 164) were included in this study. There were no statistically significant differences in general clinical characteristics, such as age, height, BMI, smoking history, drinking history, history of hypertension, history of diabetes, family genetic history, first-trimester PAPP-A level, first-trimester β-hCG level, and various second-trimester fetal ultrasound indicators, as well as most laboratory indicators between the two sets (all *P* > 0.05) (Table 1).

**Table 1:** Comparison of general clinical characteristics of pregnant women in the training set and the validation set.

Factors		Training set (*n* = 382)	Validation set (*n* = 164)	*Χ*^2^/*t*	*P*
Age (years)		27.87 ± 4.12	27.52 ± 3.98	1.830	0.067
BMI(kg/m^2^)		23.62 ± 3.08	23.41 ± 2.96	1.471	0.141
Gestational weight gain (kg)		13.48 ± 3.24	13.27 ± 3.03	1.409	0.158
Smoking history	Yes	33 (8.64)	14 (8.54)	0.070	0.791
	No	349 (91.36)	150 (91.46)		
Alcohol consumption history	Yes	17 (4.45)	7 (4.27)	0.115	0.734
	No	365 (95.55)	157 (95.73)		
History of hypertension	Yes	30 (7.85)	12 (7.32)	0.117	0.731
	No	352 (92.15)	152 (92.68)		
History of diabetes	Yes	15 (3.93)	6 (3.66)	0.013	0.906
	No	367 (96.07)	158 (96.34)		
History of pre-eclampsia	Yes	48 (12.57)	18 (10.98)	0.887	0.346
	No	334 (87.43)	146 (88.02)		
Family history of genetic diseases	Yes	33 (8.64)	14 (8.59)	0.003	0.951
	No	349 (91.36)	150 (91.41)		
First-trimester PAPP-A (mIU/L)		1.31 ± 0.38	1.28 ± 0.34	1.736	0.082
First-trimester *β*-hCG (mIU/ml)		7,560.34 ± 1,150.32	7,480.56 ± 1,120.45	1.491	0.136
Biparietal diameter (mm)		46.47 ± 3.18	46.22 ± 3.06	1.696	0.090
Head circumference (mm)		167.89 ± 10.12	167.23 ± 9.87	1.401	0.161
Second-trimester fetal abdominal circumference (mm)		144.67 ± 12.23	143.89 ± 11.98	1.368	0.171
Femur length (mm)		30.18 ± 2.49	29.97 ± 2.24	1.852	0.064
Humerus length (mm)		28.45 ± 2.23	28.32 ± 2.11	1.263	0.206
Cerebellar transverse diameter (mm)		22.21 ± 1.76	22.09 ± 1.62	1.489	0.136
Lateral ventricle width (mm)		6.28 ± 0.99	6.22 ± 0.93	1.316	0.188
Amniotic fluid index (cm)		12.03 ± 2.41	11.84 ± 2.32	1.700	0.089
Umbilical artery S/D ratio		2.86 ± 0.52	2.82 ± 0.48	1.678	0.093
Umbilical artery PI		1.06 ± 0.27	1.04 ± 0.18	1.730	0.083
Fetal heart rate (beats/min)		145.34 ± 10.05	144.96 ± 9.87	0.811	0.417

### Univariate analysis of risk factors for the occurrence of small for gestational age in the training set

In the training set, there were 72 cases of SGA infants (18.85%), and in the validation set, there were 32 cases of SGA infants (19.51%). Univariate analysis showed that there were statistically significant differences in the occurrence of SGA and indicators such as maternal age, history of pre-eclampsia, first-trimester PAPP-A, first-trimester β-hCG, Second-trimester fetal abdominal circumference, femur length, and umbilical artery PI (*P* < 0.05). In the regression model, the tolerance of each variable was >0.1, the VIF was <10, and the condition index was <30. Moreover, there was no situation where the variance proportion of multiple covariates under the same eigenvalue was >50%. Therefore, there was no collinearity among the covariates (Table 2).

**Table 2:** Univariate analysis of risk factors for the occurrence of SGA in the training set.

Factors		SGA(*n* = 72)	Non-SGA (*n* = 310)	*χ*^2^/*t*	*P*
Age (years)		29.14 ± 3.77	27.32 ± 4.03	3.493	0.001
BMI(kg/m^2^)		23.58 ± 3.04	23.78 ± 3.09	0.496	0.620
Gestational weight gain (kg)		13.37 ± 3.16	13.73 ± 3.24	0.853	0.349
Smoking history	Yes	9 (12.50)	63 (20.32)	1.676	0.195
	No	63 (87.50)	247 (79.68)		
Alcohol consumption history	Yes	5 (6.94)	13 (4.19)	0.467	0.494
	No	67 (93.06)	297 (95.81)		
History of hypertension	Yes	6 (8.33)	26 (8.39)	0.001	0.988
	No	66 (91.67)	284 (91.61)		
History of diabetes	Yes	4 (5.56)	12 (3.87)	0.100	0.751
	No	68 (94.44)	298 (96.13)		
History of pre-eclampsia	Yes	17 (23.61)	42 (13.54)	4.530	0.033
	No	55 (76.39)	268 (86.46)		
Family history of genetic diseases	Yes	8 (11.11)	26 (8.39)	0.534	0.464
	No	64 (88.89)	284 (91.61)		
First-trimester PAPP-A (mIU/L)		1.18 ± 0.32	1.36 ± 0.37	3.809	0.001
First-trimester β-hCG (mIU/ml)		7,182.54 ± 1,081.36	7,604.23 ± 1,143.42	2.847	0.004
Biparietal diameter (mm)		46.16 ± 3.08	46.52 ± 3.17	0.872	0.383
Head circumference (mm)		167.35 ± 9.97	168.03 ± 10.19	0.512	0.608
Second-trimester fetal abdominal circumference (mm)		141.23 ± 11.75	145.87 ± 12.31	2.905	0.003
Femur length (mm)		28.89 ± 2.08	29.93 ± 2.52	3.253	0.001
Humerus length (mm)		28.26 ± 2.12	27.84 ± 2.41	1.361	0.174
Cerebellar transverse diameter (mm)		22.05 ± 1.63	22.28 ± 1.69	1.047	0.295
Lateral ventricle width (mm)		6.08 ± 0.94	6.16 ± 0.97	0.634	0.526
Amniotic fluid index (cm)		11.91 ± 2.28	12.32 ± 2.43	1.304	0.192
Umbilical artery S/D ratio		2.83 ± 0.49	2.87 ± 0.56	0.558	0.576
Umbilical artery PI		1.16 ± 0.24	1.03 ± 0.27	3.754	0.001
Fetal heart rate (beats/min)		144.78 ± 9.82	145.56 ± 10.09	0.593	0.553

### Multivariate logistic regression analysis

The occurrence of SGA was taken as the dependent variable (0 = Non-SGA, 1 = SGA), and the factors with *P* < 0.05 in the univariate analysis were used as covariates for further multivariate logistic regression analysis. The results showed that maternal age, first-trimester PAPP-A, first-trimester β-hCG, second-trimester fetal abdominal circumference, femoral length, and umbilical artery PI were independent risk factors for the occurrence of SGA (*P* < 0.05) (Table 3).

**Table 3:** Multivariate logistic regression analysis of occurrence of SGA in the training set.

Factors	*B*	*SE*	Wald	*P*	OR	95%CI
Age	0.111	0.041	7.142	0.008	1.117	1.030–1.212
History of pre-eclampsia	−0.753	0.401	3.532	0.060	0.471	0.215–1.033
First-trimester PAPP-A	−1.149	0.453	6.431	0.011	0.317	0.130–0.770
First-trimester β-hCG	0.001	0.001	36.233	0.001	1.001	1.000–1.001
Second-trimester fetal abdominal circumference	−0.036	0.013	7.029	0.008	0.965	0.940–0.991
Femur length	−0.174	0.069	6.378	0.012	0.841	0.735–0.962
Umbilical artery PI	1.914	0.675	8.042	0.005	6.783	1.806–25.470

### Construction of the nomogram prediction model

**Figure 1:**
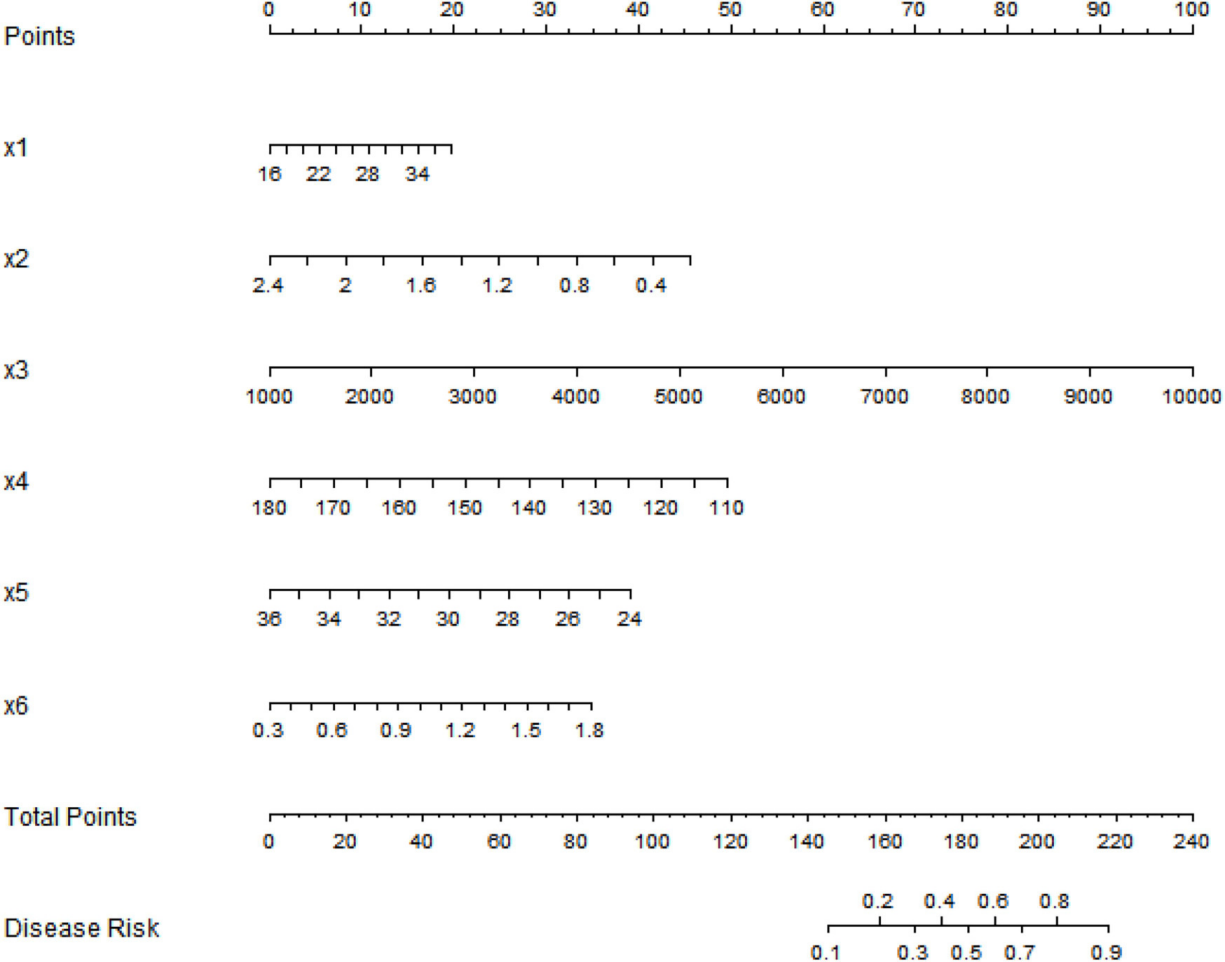
Nomogram prediction model for the risk of small-for-gestational-age infants based on multiple indicators of pregnant women. x1, Age; x2, first-trimester PAPP-A; x3, first-trimester β-hCG; x4, second-trimester fetal abdominal circumference; x5, femur length; x6, umbilical artery PI.

Based on the independent risk factors identified by multivariate logistic regression analysis, a nomogram prediction model for predicting the occurrence of SGA was constructed. Scores were assigned to each independent risk factor in the model, and the total score for predicting the occurrence of SGA was calculated, which was expressed as the probability of SGA occurrence (Figure 1).

### Evaluation and validation of the nomogram prediction model

**Figure 2:**
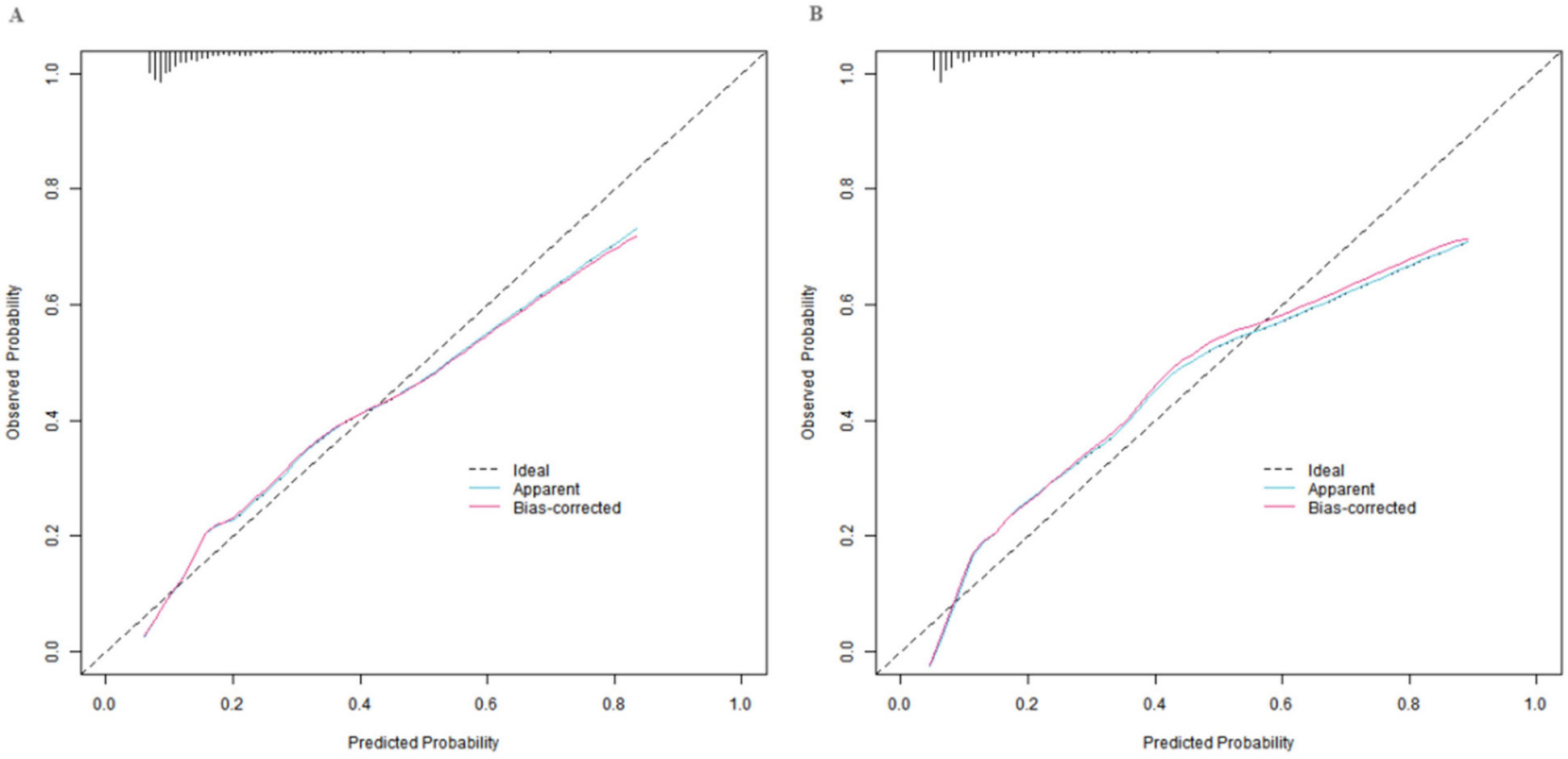
Calibration curves in the training set **(A)** and the validation set **(B)**.

**Figure 3:**
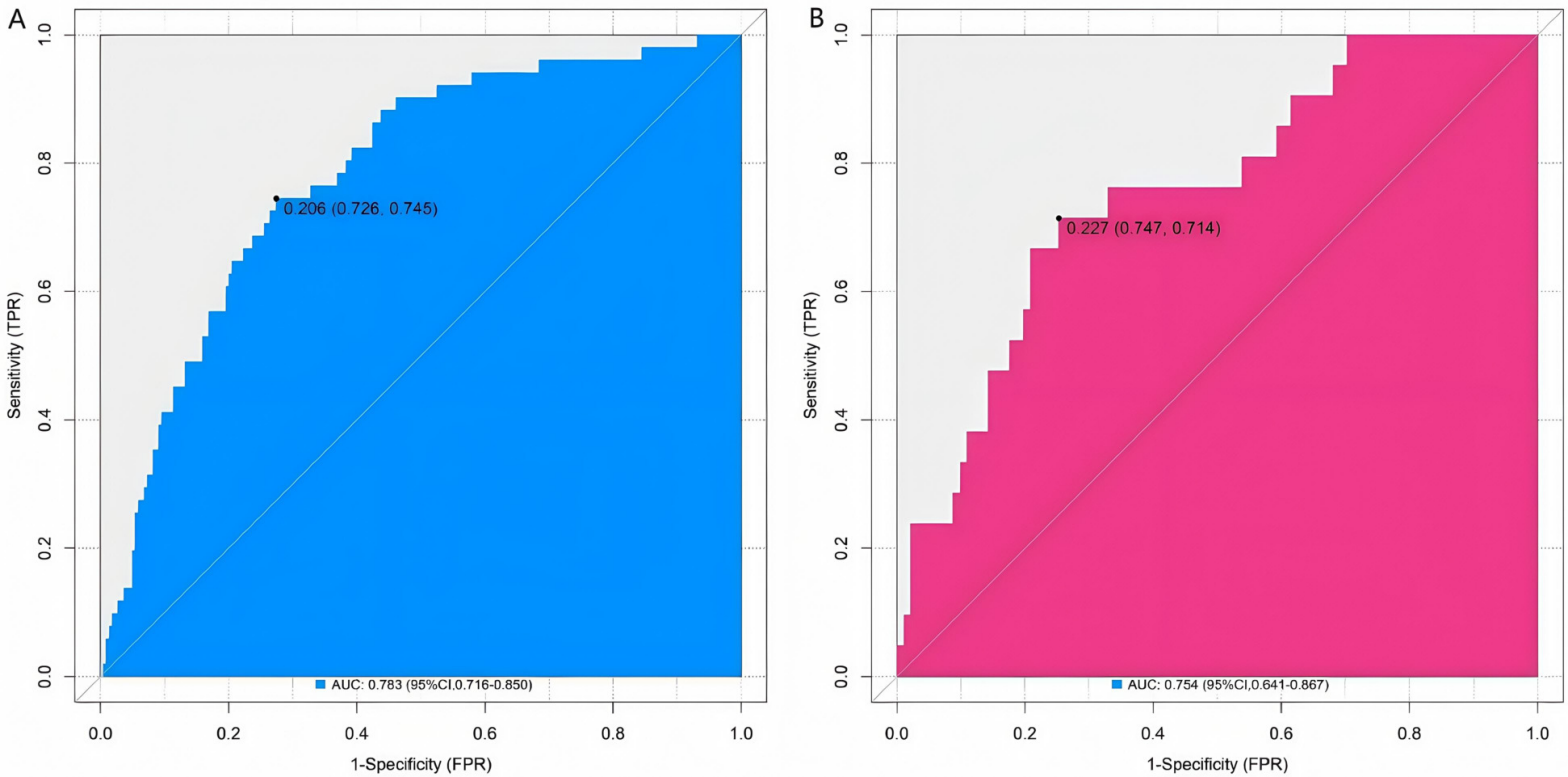
ROC curves in the training set **(A)** and the validation set **(B)**.

In the training set and the validation set, the C-index values of the constructed nomogram prediction model were 0.783 and 0.754, respectively. Further analysis through the calibration curve showed that there was a good consistency between the predicted values of the model and the actual observed values, specifically manifested as mean absolute errors of 0.128 and 0.117, respectively. Furthermore, the results of the Hosmer-Lemeshow test indicated that the *χ*^2^ values of the training set and the validation set were 15.414 (*P* = 0.051) and 15.297 (*P* = 0.053), respectively (Figure 2). In addition, the ROC curve analysis revealed the efficacy of the nomogram model in predicting the occurrence of SGA. The AUC values of the training set and the validation set were 0.783 (95% CI: 0.716–0.850) and 0.754 (95% CI: 0.641–0.867), respectively. The corresponding combinations of sensitivity and specificity were 0.726, 0.745 and 0.747, 0.714, respectively. These results indicated that the model not only performed well on the training set but also had good generalization ability on the independent validation set (Figure 3).

### Decision curve analysis of the nomogram prediction model for small for gestational age infants

**Figure 4:**
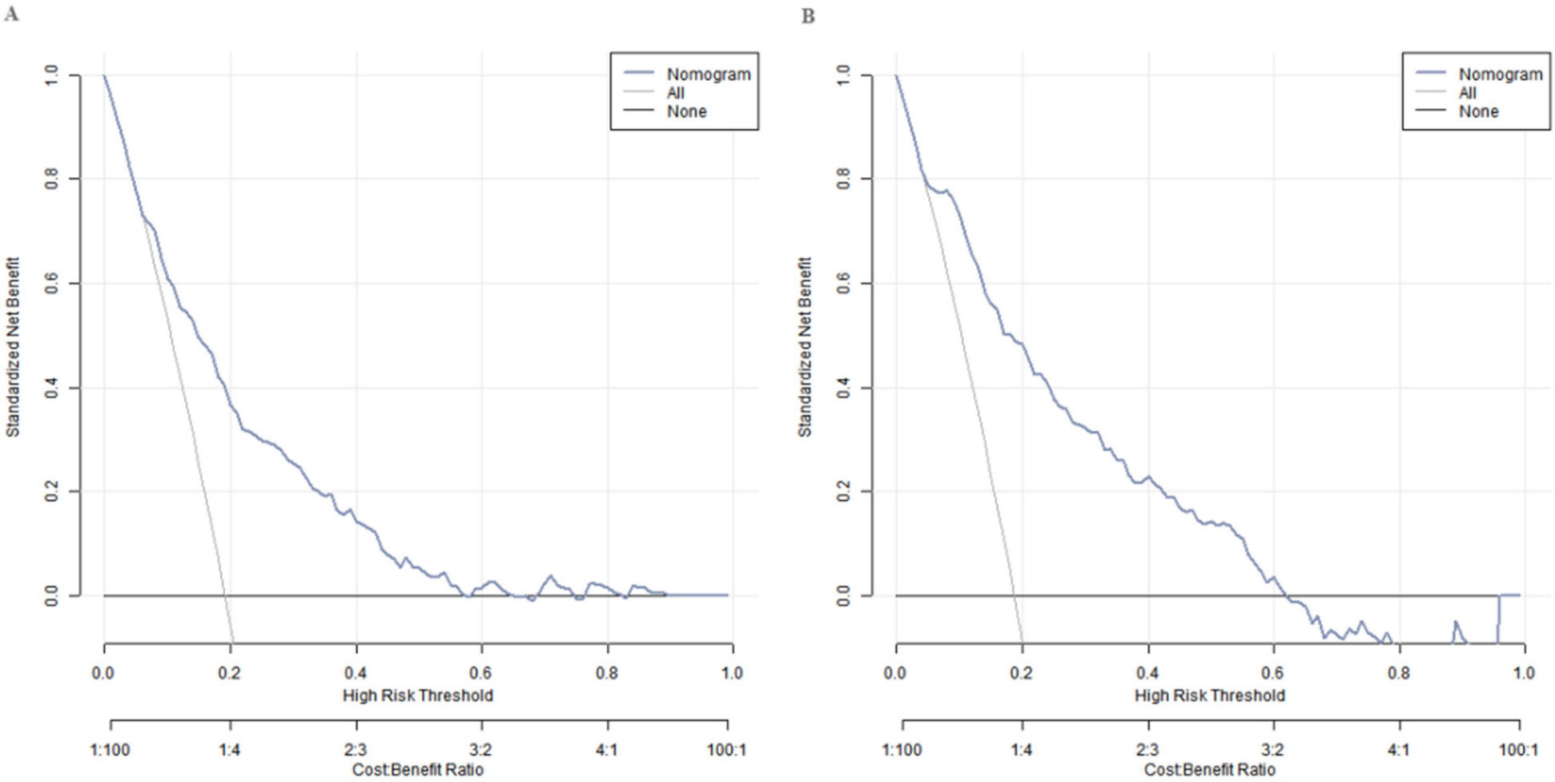
Decision curves in the training set **(A)** and the validation set **(B)**.

Decision curve analysis showed that when the threshold probability was between 0.08 and 0.65, the decision of applying the nomogram model constructed in this study to predict the occurrence of SGA had more clinical benefits compared with the decisions of pre-operatively assuming that none or all of the patients would develop SGA (Figure 4).

## Discussion

In this study, the incidence of SGA in the training set was 18.85%, and that in the validation set was 19.51%. The similar incidence rates between the two sets indicated that the research samples had good consistency and stability. Through multivariate Logistic regression analysis, it was clearly revealed that maternal age, first-trimester PAPP-A and β-hCG levels, second-trimester fetal abdominal circumference, femur length, and umbilical artery PI were independent influencing factors for the occurrence of SGA infants. This conclusion provides key clues for in-depth understanding of the pathogenesis of SGA and for clinical prevention and intervention.

As maternal age increases, a series of complex physiological changes occur in the body (9). In this process, the uteroplacental blood circulation is the first to be affected. The uteroplacental blood circulation system supplies essential nutrients and oxygen for fetal growth and development (10). Once problems occur, such as decreased vascular elasticity and slowed blood flow velocity, the efficiency of fetal nutrient and oxygen acquisition will be significantly reduced.

Both PAPP-A and β-hCG in the first trimester of pregnancy are secreted by the placenta and can sensitively reflect subtle environmental changes (11). A decrease in PAPP-A level is very likely a warning sign of insufficient placental function (12). The placenta is the site of material exchange between the fetus and the mother. Once the placental function is damaged, it cannot transfer nutrients from the mother to the fetus. Insufficient nutrient supply to the fetus will restrict its growth, thereby increasing the risk of SGA (13). Abnormal β-hCG levels, either higher or lower than the normal range, may indicate abnormal placental development and may also be an important signal of fetal growth restriction (14). When placental development is abnormal, its structure and function are affected, which directly interferes with the fetal growth environment and disrupts the originally stable and orderly development process (15).

Fetal abdominal circumference and femur length in the second trimester of pregnancy are key indicators for evaluating fetal growth and development (16). The size of the fetal abdominal circumference not only reflects the development of abdominal organs but also is an intuitive manifestation of the overall nutritional status of the fetus (17). Slow growth of the abdominal circumference may mean delayed development of fetal abdominal organs and also suggests that the fetus may have insufficient nutrient intake. Femur length mainly reflects the degree of fetal skeletal development and is an important sign of fetal physical development (18). When there is growth restriction in both fetal abdominal circumference and femur length, it is very likely that the overall development of the fetus is lagging (19). This often indicates that the intrauterine growth environment of the fetus is not ideal, and there may be problems such as insufficient nutrient supply and abnormal placental function. At this time, medical staff need to closely monitor the fetus and take corresponding measures in a timely manner to ensure the fetus's health.

An increase in the umbilical artery PI value is another signal that requires high attention, indicating an increase in the blood flow resistance of the umbilical artery (20). The umbilical artery, as an important blood vessel connecting the fetus and the mother, is a key channel for material exchange. When the blood flow resistance increases, it is difficult for nutrients and oxygen from the mother to be smoothly transported to the fetus, and the waste products produced by fetal metabolism cannot be excreted in time, which is extremely unfavorable for fetal growth (21). In the long run, it may lead to fetal growth restriction and further increase the risk of SGA.

The nomogram model showed good calibration and prediction efficacy in both the training set and the validation set. The C-index values reached 0.783 and 0.754 respectively, and the AUC were 0.783 (95% CI: 0.716–0.850) and 0.754 (95% CI: 0.641–0.867) respectively. It is worth noting that the AUC represents the probability that a randomly selected SGA case will have a higher predicted risk than a randomly selected non-SGA case, which more intuitively reflects the model's discriminatory ability. The closer the C-index and AUC are to 1, the higher the prediction accuracy of the model. The relatively high values of the two in this study indicated that the model had good prediction ability for SGA. Regarding the model's ability to identify cases, the sensitivity and specificity were 0.726, 0.745 in the training set and 0.747, 0.714 in the validation set, respectively. These values were derived based on the optimal thresholds determined by Youden's index (Youden's *J* = sensitivity + specificity − 1): the training set threshold was 0.206, and the validation set threshold was 0.227. The Youden's index optimization aimed to maximize the balance between sensitivity (ability to correctly identify SGA cases) and specificity (ability to correctly identify non-SGA cases), ensuring the model avoids excessive missed diagnoses (low sensitivity) or overdiagnoses (low specificity) in clinical practice. The current sensitivity and specificity values indicate that the model can effectively distinguish between SGA and non-SGA fetuses in clinical application, providing reliable support for clinicians to identify high-risk pregnant women and formulate targeted intervention strategies, which has certain practical value. DCA showed that when the threshold probability was between 0.08 and 0.65, the decision of predicting the occurrence of SGA using the nomogram model constructed in this study had more clinical benefits than the decision of assuming that all patients would not or all patients would develop SGA before surgery. This means that in actual clinical work, doctors can more accurately evaluate the risk of SGA occurrence based on this model and the specific conditions of patients, and then formulate more reasonable diagnosis and treatment plans to avoid over-diagnosis or missed diagnosis.

Practical implementation of the nomogram: (1) Application scenario: It is suitable for second-trimester prenatal screening (20–24 weeks), when obstetricians conduct routine fetal ultrasound examinations. (2) Operation steps: First, collect 6 indicators of the pregnant woman: maternal age (years), first-trimester PAPP-A level (mIU/L), first-trimester β-hCG level (mIU/ml), second-trimester fetal abdominal circumference (mm), femur length (mm), and umbilical artery PI. Second, find the corresponding score of each indicator on the nomogram's axis (e.g., a 30-year-old woman corresponds to a certain score for “age”). Third, sum the scores of all 6 indicators to get the “total score.” Fourth, map the total score to the “disease risk” axis to obtain the individual's SGA risk probability. (3) Clinical decision-making: If the risk probability >0.2 (optimal threshold based on Youden's index), the pregnant woman is classified as high-risk—clinicians can implement interventions such as enhanced nutritional counseling (e.g., increasing protein and calorie intake), weekly fetal growth monitoring (ultrasound), and umbilical artery Doppler follow-up to reduce adverse outcomes. The optimal thresholds for the model were determined using Youden's index: the training set threshold was 0.204 (sensitivity = 0.738, specificity = 0.704) and the validation set threshold was 0.151 (sensitivity = 0.724, specificity = 0.765). If the risk probability ≤0.2, routine prenatal care is maintained.

However, there are several limitations to this study. Firstly, the study was conducted in one hospital in Dongguan, so the nomogram may be more suitable for pregnant women in the Pearl River Delta region of China. When applied to other regions (e.g., northern China) or ethnic groups with different baseline characteristics (e.g., higher BMI), its performance may decrease. Thus, multi-center validation is needed before widespread promotion. Secondly, the model did not include maternal psychological status (e.g., chronic anxiety) or lifestyle (e.g., physical activity) — these factors may also affect fetal growth. In clinical practice, clinicians should combine the nomogram risk with clinical judgment (e.g., assessing the pregnant woman's mental state) to avoid over-reliance on the model. Finally, retrospective data may have incomplete records (e.g., missing some lifestyle information), which may slightly bias the model. When implementing the nomogram, clinicians should confirm the completeness of the 6 indicators to ensure accurate risk calculation.

In conclusion, the nomogram prediction model based on maternal baseline characteristics, first-trimester PAPP-A and β-hCG, and second-trimester fetal ultrasound indicators constructed in this study has good prediction value for SGA and provides a reference for clinical early assessment of the risk of SGA occurrence. In the future, the generalization ability of the model needs to be further verified through multi-center prospective studies, and dynamic monitoring strategies should be explored to optimize perinatal management.

## Data Availability

The original contributions presented in the study are included in the article/Supplementary Material, further inquiries can be directed to the corresponding author.
