# Supplementary figures and images for: Construction of SGA prediction model based on multi-dimensional indicators in the second trimester of pregnancy: integrating parturient characteristics, serum markers and ultrasound parameters
Source: Front Pediatr. 2025 Sep 30;13:1655615. doi: 10.3389/fped.2025.1655615 (PMC12518076; doi:10.3389/fped.2025.1655615)

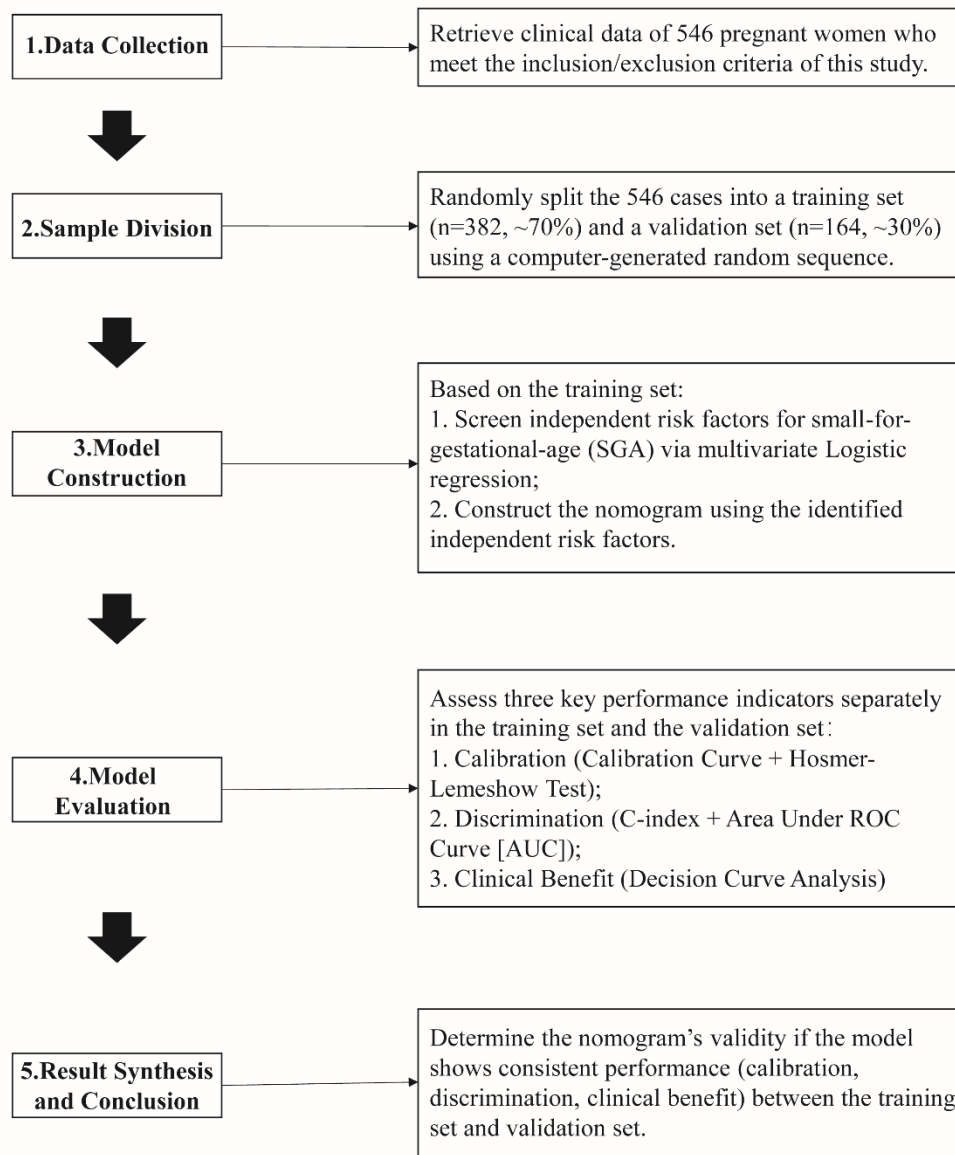

**Supplementary Figure 1** Nomogram Validation Procedure Flow Chart

Supplement: Supplementary file 1 [file Image1.pdf]
